# The Graphical Cadastre Problem in Turkey: The Case of Trabzon Province

**DOI:** 10.3390/s8095560

**Published:** 2008-09-11

**Authors:** Osman Demir, Yakup Emre Çoruhlu

**Affiliations:** Karadeniz Technical University, Department of Geodesy and Photogrammetry Engineering, 61080, Trabzon, Turkey; E-mail: osmand@ktu.edu.tr (O. D.)

**Keywords:** Turkish Cadastre, Graphical sheets, Digital cadastre, Field book

## Abstract

Cadastral projects in Turkey have been accelerated in recent years by the involvement of the private sector. These projects aim at completing the country's cadastre, along with producing bases in standards that could be a foundation for Land Registry and Cadastre Information System (LRCIS). It is possible to produce cadastral data with today's technological means. In this context, three dimensional cadastre data can be properly produced, especially in digital cadastre projects with the required point accuracy. Nevertheless this is not enough for LRCIS. The cadastre bases that have been produced so far by different methods with different scales and bases, with or without coordinates, should also be converted into digital form based on National Basic GPS Network of Turkey (NBGN) in required point-location accuracy. As the result of evaluation of graphical cadastre bases produced without coordinates, actual land measurements, and information obtained from sheets and field book data together, it was found out that there are significant base problems in the graphical maps. These bases, comprising 20% of Turkey's cadastre constitutes the most important bottleneck of completing the country's cadastre. In the scope of this paper, the possibilities of converting the field book measurement values of graphic cadastre bases into digital forms in national coordinate system by comparing them with actual land measurements are investigated, along with Turkey's Cadastre and its problems.

## 1. Introduction

The research projects for the Land Registry and Cadastre Information System were started in Turkey in the 1990s. However, the actual progress of these projects shows that the targeted aims have still not been realized because of existing problems, especially with cadastre bases. Three important tasks that have to be realized were determined as the result of these studies. These tasks are:

1. Reconstituting the cadastre bases produced so far by applying required transformation and adaptation steps to the national coordinate system in digital format.
2. Producing new cadastral maps in the national coordinate system with required accuracies.
3. Setting up a suitable geodetic network to produce these bases.

Only 29% of the digital cadastre bases produced so far possess the properties needed to be bases for information systems, therefore this is an important quality problem for the country's cadastre [1, 5].

Other countries in the world that have maintained some form of cadastral mapping are now proceeding to a full digital representation of these cadastral maps with the ultimate aim of having country wide coverage at an accuracy level consistent with current Geographic Information Systems (GIS) and surveying technology. GIS have shown the economic and information benefits of integrating the data sets and functions of the mapping, land titling and land management institutions. Also, in each of these countries the current status and future directions of digital cadastral databases (DCDB) in any juristidiction depends very much on the historical origin of the cadastral systems and the land related institutional structures (or current restructuring). In contrast to the varied paths to the present digital cadastral maps of any country the current problems and required solutions are surprisingly similar worldwide [12, 13].

The Republic of Turkey was founded in 1920. In the period that followed there were many reforms including Cadastre which started on a small scale in 1924. Till today, cadastral works have been carried out under different regulations and with various technological tools. The first national cadastral target was specified in the ‘First Five Year Development Plan’ in 1963 and it was planned that the Turkish cadastral works would be completed in twenty years. However, to date this plan has not been realized. In addition, existing cadastral sheets have been produced using different coordinate systems, scales, surveying methods, position accuracy and sheet types. Almost 20% of these sheets have been produced on paper bases using graphical method having no coordinate values. Most of these sheets have incompatible field-sheet-measurement values. The rural to urban transformation is another cause of the inadequacy of the sheets, so today, most of the Turkish cadastral maps have no applicability to the field.

In this paper, the Turkish cadastral system, the methods used in the cadastral work, position accuracies of the cadastral bases, the existing situation of the cadastral works and the problems of Turkish graphic cadastre are described in detail.

## 2. The Turkish Cadastre Process

Turkish cadastre is based on the initial cadastral works carried out during the Ottoman Empire period. With some exceptions, all of the lands within the Ottoman Empire were under state ownership. While these lands' basic ownership and supervision rights belonged to the state, the usage rights belonged to individuals. This system continued until the late nineteenth century. With the Land Law in 1858, the transition to private ownership system began [7-10]. After the foundation of the Republic of Turkey, private ownership policy continued and was legislated by Civil Law and Cadastre Law transferred from western countries. In the Ottoman Empire, because most land was under state ownership, written cadastre data was seen as sufficient. However, after the foundation of Republic of Turkey, due to the continuing shift towards private ownership, modern cadastral works based on line cadastre were started to provide secure land ownership. This historical background is necessary in order to understand the legal and technical process of the Turkish cadastral system from 1924 to date.

### 2.1 The Legal Process

After the foundation of the Republic of Turkey, cadastral works were initiated in 1924 with Law No. 474. This law provided for the determining of real estates' owners' incomes, values and geometrical situations in some counties and provinces. In these studies, instead of maps, sketches and their supporting documents were prepared as a single copy, so these works cannot be considered proper cadastre works. In 1925 under Law No. 658 the cadastre organization was constituted under the General Directorate of Land Registry. The purpose of this organization was determining property and boundaries of real estates and their classification in terms of position and economic situation. Cadastral work was started in some big cities. In 1934, the Cadastre and Land Registry Law was put into practice and then, regulations were prepared in 1935. Cadastral work had been carried out based on this law, especially in urban areas. In 1950, the Land Registry Law (No. 5602) was put into practice to speed up cadastral work in rural areas. Known as ‘land cadastre’, this law was changed in 1964 and 1966 and became the Land Registry Law (No. 766). Cadastral work had been carried out in urban and rural areas under two different laws until 1987. The Cadastre Law (No. 3402) was put into practice to eliminate the problems originating from having two different laws and to gather whole cadastral regulations into one law. However, in the forest areas the cadastral works are still carried out under a different law (No. 6831) undertaken by General Forest Directorate [5].

### 2.2 Technical Process

From a technical perspective, Turkish cadastre has two different cadastral periods. These are the written and linear cadastre periods, respectively.

#### 2.2.1. Written Cadastre Period

Written cadastre means that cadastral information is expressed in words instead of linear data. In the written cadastre period, technical infrastructure was not sufficient for the visualization of the position based data and also there were not enough technical staff to carry out this process. In the written cadastre, real estate boundaries were identified in the land title document with the features to the north, south, east and west of the property, for example there is a village road to the south, a river to the north. Such studies had been carried out from Ottoman Empire period to 1934. These documents are still used as property evidence in the areas where linear cadastral works have not yet been carried out [6].

#### 2.2.2 Linear Cadastre Period

With the introduction of arithmetic and geometric applications, cadastre gained a technical perspective. In this context, the Turkish linear cadastral works were initiated in the early 1990's. The linear cadastre period can be grouped into four categories: graphic, classic, photogrammetric and electronic tachometry methods.

**Figure 1.**
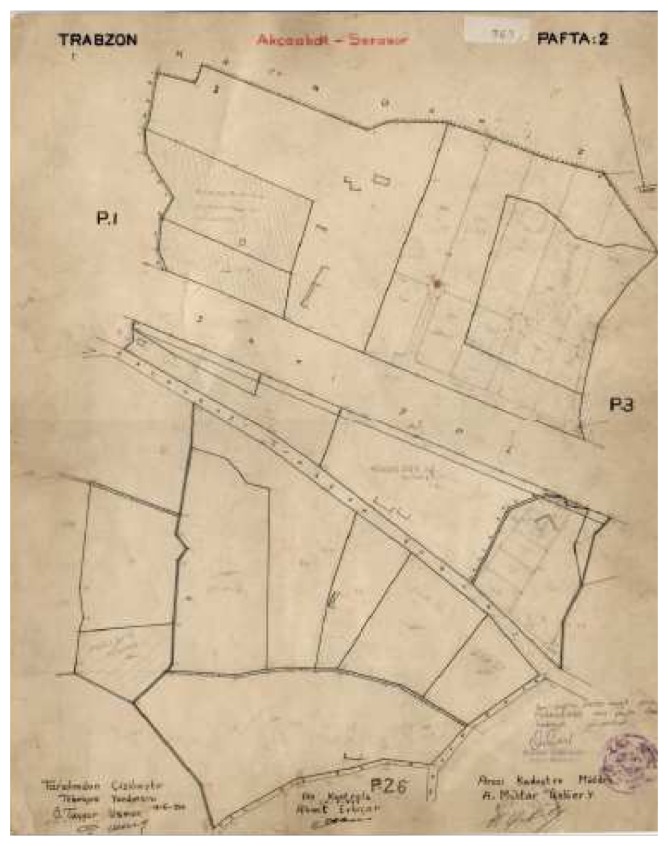
A sample cadastre map produced by the graphical method.

a) Graphical Method: In Turkey, this was the first type of cadastral work; parcel corner coordinates were surveyed with chain surveys based on the polygons which exist on the ground but having no coordinate values (Figure 1). Thus, the parcel surveying values derived from this method generally have no control procedures and the bases produced by graphical method do not have sufficient quality for today's requirements. In other words, most of these bases have ground-sheet inconsistencies. The bases produced by graphical method constitute 20% of the whole cadastral bases in Turkey.
b) Classical Methods: These are generally orthogonal and tachometric methods. Those works carried out before 1968 were based on polygons, later they were based on localized land surveying triangulations. While, the orthogonal method was used in urban areas under Law No. 2613, the tachometric method was used in rural areas under Law No. 766. After 1974, based on national land surveying triangulation network these surveys became widespread. The obligation to produce cadastral bases in national coordinate system was put into practice after 1993.
c) Photogrammetric Method: After 1950, this method was frequently used in order to accelerate cadastral work. The photogrammetric method was used in the areas where land cover and topography is suitable. Therefore, intensive land titling work was carried out in the interior parts of the country. As a result, generally 1/5,000 scaled cadastral maps were produced. These bases have lost their validity especially in the areas transformed from rural to urban use because of the low accuracy of the positions.
d) Electronic Tachometry Method: Because of its speed and sensitivity, this method was used in cadastre as a ground surveying method after 1985. After the integration with computer technology, electronic tachometry has become very effective tool in cadastral works especially in the rough, plant covered and urban areas where using photogrammetric techniques are inappropriate. Digital cadastral works using this technique still are being used [6].

The following table summarizes all the cadastral maps produced by using different production methods (Table 1).

### 2.3. Institutional process

In the Turkish Republic, the institutional structure for cadastral system was organized in 1936. This structure was constituted as ‘Land Registry’ and ‘Cadastre’ directorates. While land registry directorates have carried out the assembly and sustent of land registries, cadastre directorates have carried out cadastral works and its sustent since 1936. On the other hand, in addition to these organizations, the technical aspect of cadastral projects could be contracted to the private individuals or legal entities in appropriate areas according to the ‘Cadastre and Land Registry Law’ dated 1934. The same issue become very relevant in the 1980's and then the same article was added to the ‘Cadastre Law’ dated 1987. However, because of lack of technical personnel and devices, this article was not been executed until 2004. At that time, ‘direct income support’ has come into use for farmers as an agricultural policy in Turkey. However, there was no accurate graphical data for agricultural parcels in some regions. Cadastre data became an urgent need for these areas, and as a result of this situation, the article has put into practice. Only technical parts of cadastral projects were contracted to private surveyors in some areas as pilot projects [3]. Today, these projects are increasingly carried out by the private sector.

### 2.4. Realization Ratio of Turkish Cadastral Works

The total cadastral area of Turkey was declared to be 430,000 square kilometer in the ‘Third Five Year Development Plan’ in 1973. 410,000 km^2^ is in rural areas, the remaining part in urban areas. Almost 85% of this cadastral target had been carried out by the year 2007 and 40,736,511 parcels were registered in the land registry books (Table 2).

### 2.5 The Turkish Cadastre Problems

In the Turkish cadastre, many of the existing plans and documents are inadequate, because they are lacking technical standards or worn-out. Updating cadastral plans and related documents is a vital issue in a cadastral system and this issue is more important for countries such as Turkey that are undergoing rapid changes in real estate distribution. In this context, the other important problem for Turkey is that most of the changes in real estate have not been updated in plans and associated documents.

On the other hand, a majority of Turkish cadastral maps are in local coordinate systems, while the remander are in the national coordinate system. Maps have been produced and have been updated in the national coordinate system since digital cadastre was introduced in Turkey [2], so there is an additional need for transformation of old cadastral maps (approximately 93%) into the national coordinate system.

The graphical method was widely used in land titling works in Turkey till the 1960's, then after that time, it was used less regularly. In this work, surveying procedures were not based on land triangulation points and polygon stones were not used in many areas. Today, it is almost impossible to find and use the points which were used in these studies. As a result, application of these bases in the field is very difficult. There are also important geodetic point problems in the constitution of new cadastral bases in the national coordinate system and for transforming of existing bases to this system [5]. Nowadays, because of the technical deficiencies of graphical cadastre bases that are legally in effect, delays are being experienced in some important engineering projects and this further causes some socioeconomic problems. In this context graphical cadastre bases constitute an important problem of Turkey's cadastre.

## 3. The method for graphical cadastre measurement

At the beginning the graphical cadastre sheets were produced based on such methods that could be described as primitive. The essential technical features of these sheets, produced for rural areas, are:

- There was no land surveying triangulation in the country.
- Closed control points were created. Closing errors in these polygons were distributed to the points in the whole graphics.
- Detailed measurements were carried out with a tachymeter. The angles on the other hand were formed as a complete series.
- The villages were done with the prismatic method.
- Drawing both polygon points and detail points were realized with the polar method.
- Since some bases used were aluminum, some on good quality of cardboard, and some others on bad quality of cardboard, the overall accuracy of these graphic sheets are extremely low.
- It is no longer possible to find in the field most of the control points of the maps.

The cadastral works realized on rural areas in the past, today, mostly inside the urban or city development areas. Evaluated in this respect, the graphical cadastre bases covers the areas with very high land values as well. Therefore, in these regions, cadastre based problems stemming from graphical cadastre are being experienced in engineering or city infrastructure applications.

**Figure 2.**
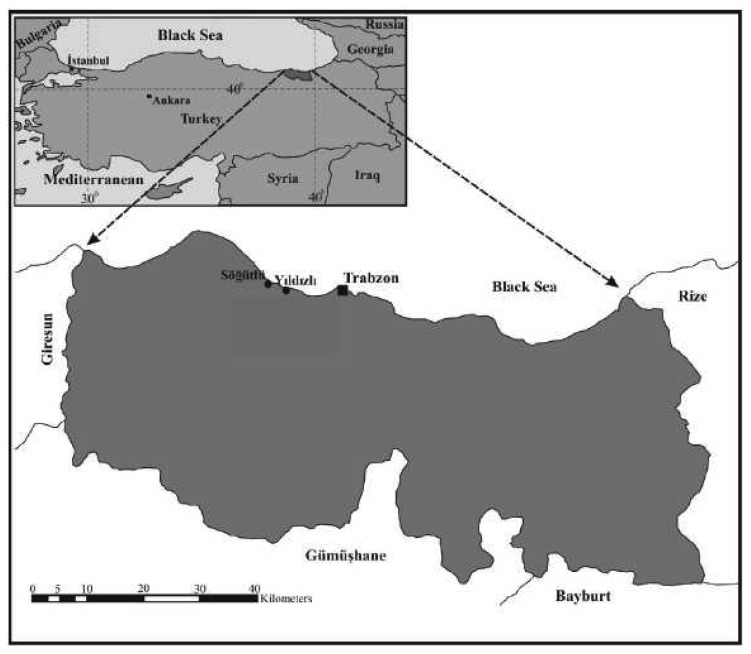
Application area.

The Yıldızlı and Söğütlü regions of the Akçaabat district in Trabzon province were chosen as the application area. Cadastral works in these regions were done in 1954 using the graphical cadastre measurement method. Regions that were rural at that time are now included in the urban or urban development areas (Figure 2). Public improvements were realized in a large part of Yıldızlı region by performing lot and land readjustment to meet the increasing land demand. Consequently, land values have been increasing in the region day by day, and keeping the cadastre bases up-to-date applicable gains even more importance.

### 3.1. Digitizing the graphical cadastre bases

**Figure 3.**
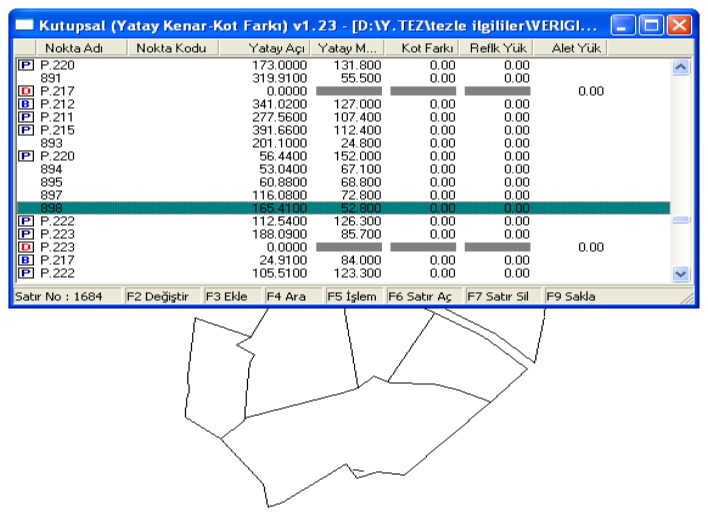
Digitizing parcels based on field book values.

The actual cadastral data obtained from Directorate of Land Registry were evaluated in this respect. First of all, the cadastre sheets were regenerated graphically by transferring field book observation on which the cadastre measures were based into a computer. In order to do this, before all else, polygons used in parcel measurements in application area are transferred into computer by using observation values. Since the graphical cadastre bases are without coordinates, after defining coordinates in any system, the measured points were transferred into digital environment The cadastre parcels were formed in the digital environment by transferring observation values of parcel corners into a digital environment and by linking them according to field book sketchs (Figure 3).

### 3.2. Problems in digitizing graphical cadastre sheets

The suitability of the produced cadastre base was tested by comparing it with the actual sheet. At this phase some important problems were faced. The comparisons were perfomed based on cadastre blocks. While one to one matches occurred for some cadastre blocks located in the same sheet, no matches were found for some other cadastre blocks in the same sheet. The cadastral sheet, produced as a result of drafting of observation values by using protractor and ruler, was found to not be in harmony with the base produced by using the same observation values. In this aspect, important drafting errors, shifts, turns, and even scale errors were determined.

### 3.3. Verification analysis of produced digital cadastre data

The most important data in determination of accuracy of created cadastre bases are coordinate values. The size of differences in coordinates representing the same points gives the accuracy of digital cadastre bases. In this sense, given the following;

y_a_, x_a_: the coordinates obtained from the land by actual measurements,
y_o_, x_o_: the coordinates obtained by digitizing the field books,
x_p_, y_p_: the coordinates obtained from sheets by digitization,
ε_x_, ε_y_ errors are calculated as in the formula(1,2,3) below with the assumption that for a parcel's corner point, the digitized coordinates of the point are in the same direction and with the approach that there are real values of coordinates whose land coordinates are digitized;
  (1) $$\varepsilon_{\text{y}}={\text{y}}_{\text{a}}-{\text{y}}_{\text{o}}\;,\varepsilon_{\text{x}}={\text{x}}_{\text{a}}-{\text{x}}_{\text{o}}$$
  (2) $$\varepsilon_{\text{y}}={\text{y}}_{\text{a}}-{\text{y}}_{\text{p}}\;,\varepsilon_{\text{x}}={\text{x}}_{\text{a}}-{\text{x}}_{\text{p}}$$
  (3) $$\varepsilon_{\text{y}}={\text{y}}_{\text{o}}-{\text{y}}_{\text{p}}\;,\varepsilon_{\text{x}}={\text{x}}_{\text{o}}-{\text{x}}_{\text{p}}$$

By using these values, from the equations (4) below

(4) $${\text{m}}_{\text{x}}=\sqrt{\frac{\left[\varepsilon_{\text{x}}\varepsilon_{\text{x}}\right]}{n}},\;{\text{m}}_{\text{y}}=\sqrt{\frac{\left[\varepsilon_{\text{y}}\varepsilon_{\text{y}}\right]}{n}},\;{\text{m}}_{\text{p}}=\sqrt{{\left[m_{x}\right]}^{2}+{\left[m_{y}\right]}^{2}}$$

the mean error in x direction mx, the mean error in y direction my and mean square error mp are calculated. By assessing εy, εx measurement differences for every sheet separately, whether they are suitable for normal distribution is analyzed by constructing error distribution histograms by employing χ2 test [14, 4, 8].

The data sources can be analyzed by comparing parcel corner points obtained from land-field books. The displacement value of parcel corner points is calculated as in formula (5) from the differences of the points given in equations (1), (2) and (3)

(5) $$\varepsilon_{i}=\sqrt{\varepsilon_{x}^{2}+\varepsilon_{y}^{2}}$$

The mean error of εi is calculated as in formula (6) where ma, mp, are the mean error of data obtained from land and sheet respectively, and m0 is the mean error obtained from field book.

(6) $$\begin{array}{c} m_{\varepsilon }=\sqrt{m_{o}-m_{p}}\;\text{Field Book}‐\text{Sheet}\\ m_{\varepsilon }=\sqrt{m_{o}-m_{a}}\;\text{Field Book}‐\text{Land}\\ m_{\varepsilon }=\sqrt{m_{a}-m_{p}}\;\text{Land}‐\text{Sheet} \end{array}$$

The result of the calculation in Equation (7) below is the size of the test.

(7) $${\text{T}}_{\text{i}}=\frac{\left|\varepsilon_{i}\right|}{m_{\varepsilon }}$$

The theoretical standard deviation is a value that describes the main group. The numerical value of this can rarely be known. Consequently, if the real error ε is standardized by dividing it by the experimental standard deviation rather than theoretical standard deviation, the random variable t_f_ is obtained.

(8) $$t_{f}=\frac{\varepsilon }{s}$$

The experimental standard deviation is calculated as in the Equation (9) from the real errors given that is the number of unknowns [8, 4].

(9) $$s^{2}=\frac{\left[\varepsilon \varepsilon \right]}{n},\;s^{2}=\frac{\left[\nu \nu \right]}{n-u}$$

t-table value is compared with T value. According to the result of comparison;

✓ if *T_i_*〈*t*_*f*,1_*_α_*, the variation in parcel s corner points is harmonious,
✓ if *T_i_*〉*t*_*f*,1_*_α_* , the variation in parcel s corner points is not harmonious.

A total of 395 parcel corner points were assessed in the application area as described above. Accordingly, the obtained results are given in the table below. From these data m_p_, called root mean error of an obtained point, was calculated (Table 3).

### 3.4. The comparison of land values of produced digital cadastre bases

The parcel areas in application regions were calculated by employing coordinate data obtained from each of digital cadastre bases constructed by data of sheet, land and field books. Each of the calculated areas then was compared with the areas shown in the title deed. Approximately 65 % of title deed lands in graphical cadastre bases are found to be erroneous above a certain error limit (Table 4).

### 3.5. Findings related with Shifts and Rotations on Cadasral Maps

**Figure 4.**
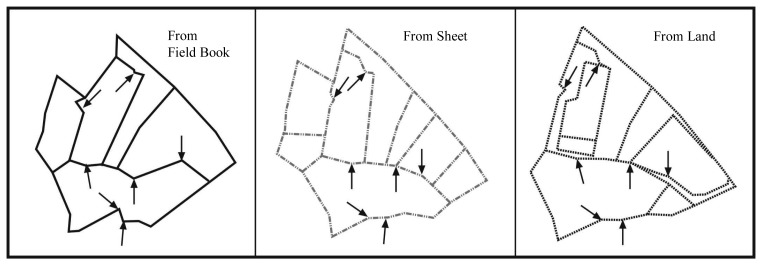
The superposition of cadastre bases created from land, sheet and field book values for the same parcels.

**Figure 5.**
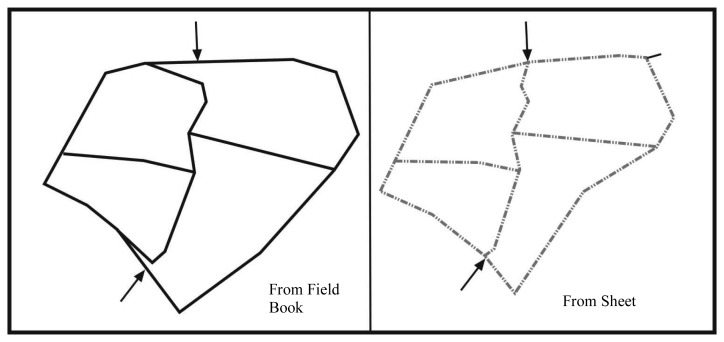
The differences in parcel geometry created according to the field book-sheet-data that belong to the same parcels.

The shifts, shrinking, and rotations occurred in cadastre parcels are more clearly seen when created digital cadastre bases are compared one on the top of the other after transformation into the national coordinate system in a CAD environment. The actual mean square error values in digital cadastre bases are given in the previous page. Here the aim is to give a graphical representation of the reflection of errors to the parcels. In order to do this, the shifting ways of cadastre bases produced on the parcel or city sheet basis are given along with discrepancies in the geometry of parcels in the figures below [10, 11, 13, 4] (Figures 4, 5).

### 3.6. Findings related to statistical test results on digital cadastre bases

Three different cadastre bases, created separately by using land, sheet and field book cadastre observations, were statistically assessed. In order to do this firstly the coordinate differences of the points in each of three systems were calculated. Accordingly statistical data that belong to differences of processed points in x and y directions were obtained (Table 5).

One Sample Kolmogorov - Smirnov Z nonparametric statistical test method was applied to find out the confidence interval of the created digital cadastre data and if they are in normal distribution. The related findings are given in the table below (Table 6).

The results of statistical results related with created digital cadastre bases are given below separately, according to the methods. In these figures histograms of frequencies and normal distribution curves on the histograms are shown. Normal P-Plot graphic that shows cumulative rates of a variable against the cumulative rates of normal distribution is depicted next to each of the histograms. The goal here is to determine where the normal distribution curve of variables is gathered.

**Figure 6.**
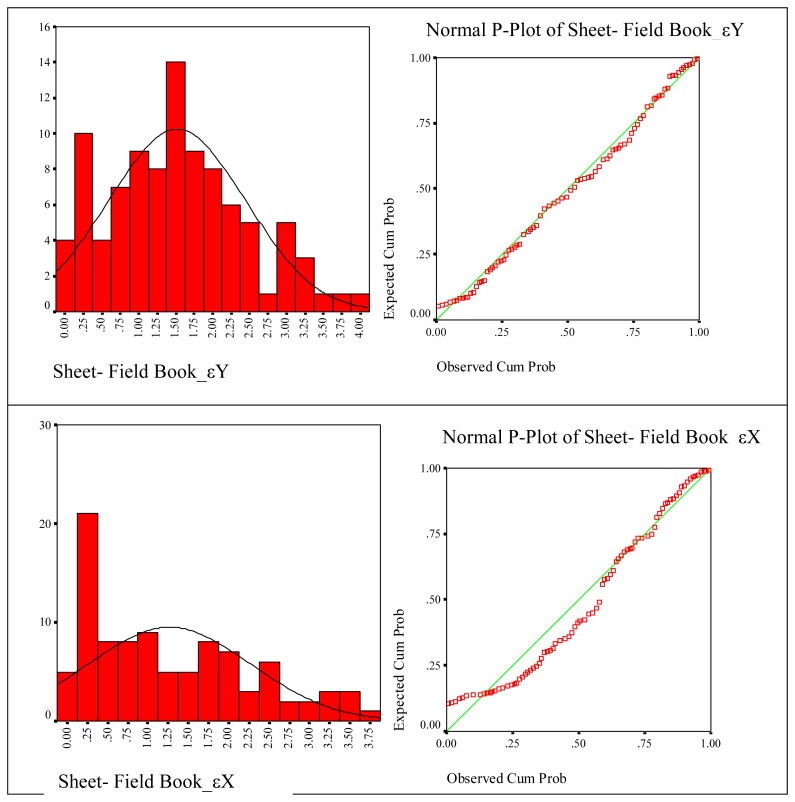
Coherence of coordinate differences of sheet-field book to the normal distribution.

Taking into account digital cadastre data created according to sheet and field book data, it was determined that ε_Y_ distribution suits for normal distribution with 95 % confidence, its statistical values are P=0.900, *χ*^2^ = 0.571. Sheet- field book ε_X_ distribution suits for normal distribution with %95 confidence and its statistical values are P=0.175, *χ*^2^ =1.103 . The approximation of variables to normal distribution curve is seen according to P-Plot test (Figure 6).

**Figure 7.**
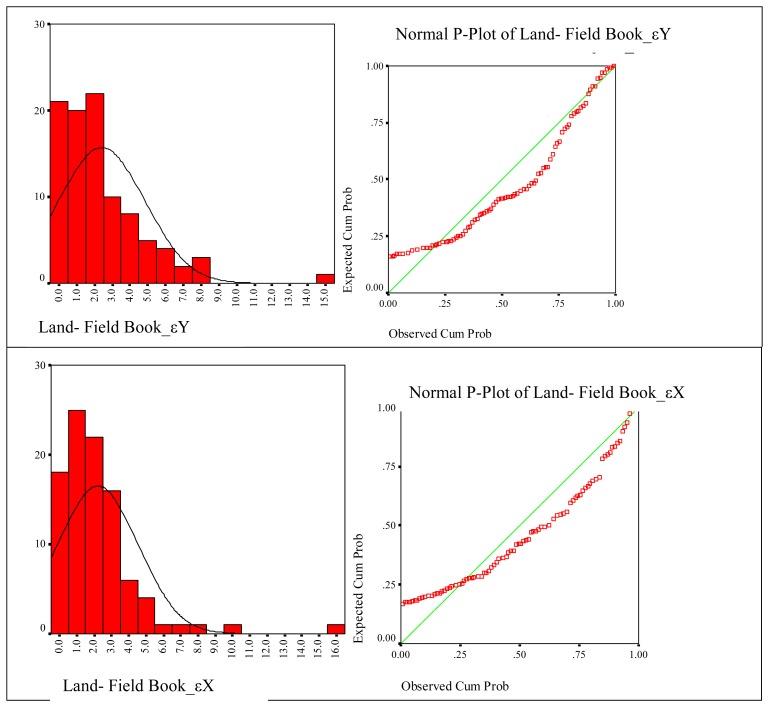
Coherence of coordinate differences of land-field book to the normal distribution.

Considering, on the other hand digital cadastre data created by using land and field book data, the εY distribution does not fit a normal distribution with 95% confidence and its statistical values are P=0.013, χ2=1.588; land-field book εX distribution does not fit a normal distribution with 95% confidence and its statistical values are P=0.008, χ2=1.669. The approximation of variables to normal distribution curve is seen according to P-Plot test (Figure 7).

**Figure 8.**
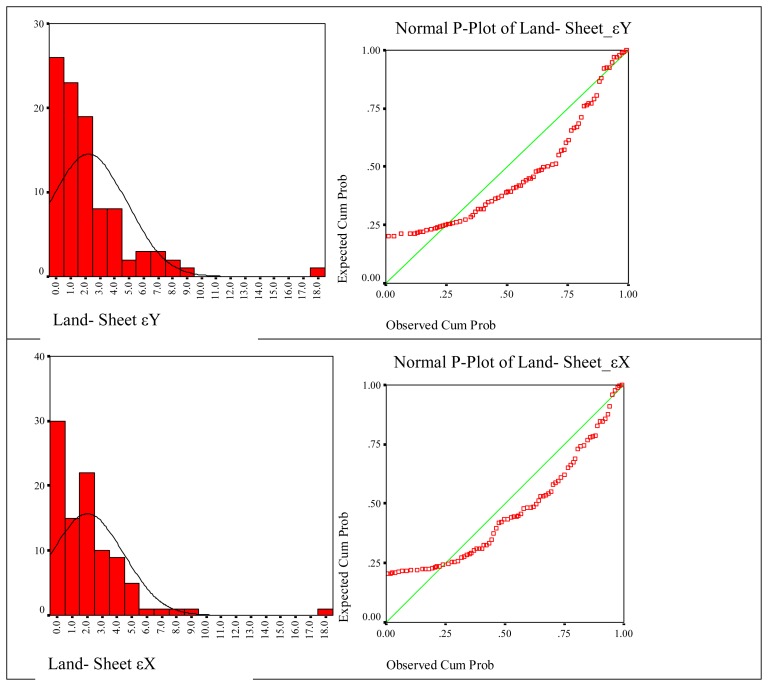
Coherence of coordinate differences of land-sheet to the normal distribution.

Considering finally the digital cadastre data created by using land and sheet data, land-sheet εY distribution does not fit a normal distribution with 95% confidence and its statistical values are P=0.001, χ2=1.972, land-sheet εX distribution does not suit for normal distribution with 95% confidence and its statistical values are P=0.001, χ2=2.007. The approximation of variables to normal distribution curve is seen according to P-Plot test (Figure 8).

The statistical tests conducted revealed that the cadastre bases produced at the beginning of the work with errors far bigger than required point location sensitivities. The tests also suggest that 40 of 395 points, which is approximately %40, are correct measurements. This situation proves that graphical cadastre data were still produced inaccurately at the beginning.

## 4. Conclusions

It is seen that graphical cadastre sheets were not produced with required accuracy and the rules that should have been obeyed were significantly violated. In this respect it was determined that there were still problems at the beginning of cadastre and these problems were reflected in the parcel corner points far above the acceptable error limits. Accordingly, the following situations that could affect the sensitivity of cadastre bases were determined.

- Because of the primitive techniques used and the wear, shrinking, and defacement problems of the sheets, together with location and drafting errors, edge-matching problems, shifts, and overriding were determined. The errors calculated according to sheet-field book were naturally smaller than the others. The largest number of coherent points were produced from sheet-field books as a result of statistical test results. But, it was seen that the rate of coherent points to the total data is only 10%, which suggests that graphical cadastre data at the beginning were still produced inaccurately.
- A significant part of graphical cadastre bases covers urban development regions. In these regions where the value of land is extremely high, parcels are sold according to the areas shown in the title deeds. At this point it was determined that 65% of the parcel areas in the application areas are erroneous. In these regions, stopping parcel sales until the cadastre bases can be produced with the required point accuracy could prevent possible socioeconomic problems.
- The average quadratic error of a point was determined to be m_0_=+/- 4.70m in created digital cadastre bases when land and field book data are taken into account. Therefore it is extremely important that use of those cadastre bases technically not right and should be banned legally, and correct and up to date cadastre bases should be created by carrying out cadastre works in these regions again. Considering that graphical cadastre bases cover 20% of Turkey's cadastre, this reveals the necessity of conducting cadastre projects in a large part of the country. This further suggests that a second cadastre is no longer avoidable for our country.

## Figures and Tables

**Table 1.** Turkish cadastral maps according to production methods [15].

**No**	**Production Method**	**Number**	**%**
1	Graphic	110710	0,20
2	Polar	127118	0,24
3	Orthogonal	61271	0,11
4	Photogrammetric	87254	0,16
5	Digital	154008	0,29

Total: 5 different map production methods		540361	100.00

**Table 2.** Realization of the Turkish cadastral works [6].

**Period of cadastre works**	**Number Parcels Produced**
1925-1984	22458928
1985-1989	3100502
1990-1994	3473851
1995-2000	3288483
2001-2004	2850622
2004-2007	5564125

Total Number Parcels Produced	40736511

**Table 3.** The summary table of location sensitivity.

**Parcel Corner Number of points**	**Location sensitivity**	[*ε*_x_*ε*_x_]	**m_x_(m)**	⌊*ε_y_ε*_y_⌋	**m_y_(m)**	**m_p_(m)**
**395**	Field book measure-sheet	1262.79	1.788	1038.85	1.622	2.415
**395**	Land-Field book measure	4655.26	3.433	4064.55	3.209	4.700
**395**	Land-Sheet	4696.03	3.448	3974.32	3.172	4.685

**Table 4.** The parcel's area differences in graphical cadastre bases.

**Type of parcel area**	**Error limits**					
	**In the**		**Out of**		**Total**	
	**P.S.**	**%**	**P.S.**	**%**	**P.S.**	**%**
**Land –Title deed**	24	36	43	64	67	100
**Land - Sheet**	18	27	49	73	67	100
**Land - Field book**	11	14	56	85	67	100

**Table 5.** Statistical test results of digital cadastre areas.

**Statistical Values**	**%**	**Sheet –Field Book (m)**		**Land – Field Book(m)**		**Land – Sheet (m)**	
**Differences of Coordinates**		**ε_y_**	**ε_x_**	**ε_y_**	**ε_x_**	**ε_y_**	**ε_x_**
**Number of Sample**		395	395	395	395	395	395
**Mean**		1.5171	1.2666	2.4089	2.2085	2.2145	2.0142
**Standard Deviation of the mean**		0.09526	.1026	.2485	.2365	.2688	.2493
**Median**		1.4700	1.0500	1.8950	1.7550	1.4850	1.6100
**Standard Deviation**		0.9334	1.0054	2.4352	2.3174	2.6338	2.4427
**Variance**		0.8711	1.0109	5.9300	5.3706	6.9369	5.9668
**Minimum**		0.01	0.01	0.00	0.00	0.01	0.00
**Maximum**		3.98	3.75	15.00	16.17	18.00	17.96
**Percenteges**	10	0.2170	0.1700	0.2070	0.2350	0.1200	0.1270
	20	0.6880	0.2700	0.4440	0.5280	0.2920	0.2140
	30	0.9720	0.4830	0.7250	0.8510	0.5340	0.4200
	40	1.2700	0.7760	1.4140	1.2680	0.9780	0.8180
	50	1.4700	1.0500	1.8950	1.7550	1.4850	1.6100
	60	1.6700	1.4620	2.1420	2.1800	1.8880	1.9040
	70	1.9170	1.7790	2.7390	2.5400	2.3070	2.4910
	80	2.3400	2.1960	4.1680	3.3320	3.6080	3.3900
	90	2.9030	2.8020	5.6650	4.5050	5.9520	4.5030

**Table 6.** Summary table of One Sample Kolmogorov - Smirnov Z nonparametric statistical test.

**Statistical Values**		**Sheet-Field Book**		**Land-Field Book**		**Land-Sheet**	

		**ε_y_**	**ε_x_**	**ε_y_**	**ε_x_**	**ε_y_**	**ε_x_**
**Number of Samples**		395	395	395	395	395	395
**Normal Parametres**	Mean	1.5171	1.2666	2.4089	2.2085	2.2145	2.0142
	Stan. Dev.	.9334	1.0054	2.4352	2.3174	2.6338	2.4427
**Ekstreem Differences**	Final Value	0.058	0.113	0.162	0.170	0.201	0.205
	Pozitive	0.058	0.113	0.162	0.151	0.194	0.148
	Negative	-0.053	-0.106	-0.161	-0.170	-0.201	-0.205
**Kolmogorov Smirnov Z**		0.571	1.103	1.588	1.669	1.972	2.007
**Asymp.Sig. (2-tailed)**		0.900	0.175	0.013	0.008	0.001	0.001
